# Survival Outcomes in EGFR‐Mutant Non‐Small Cell Lung Cancer With Brain Metastases: Kaplan–Meier and Cox Regression Analyses Across Treatment Stages

**DOI:** 10.1111/crj.70085

**Published:** 2025-05-27

**Authors:** Haoran Qi, Qiang Qiao, Xiaorong Sun, Ligang Xing

**Affiliations:** ^1^ Department of Radiation Oncology Shandong Cancer Hospital and Institute, Shandong First Medical University and Shandong Academy of Medical Sciences Jinan Shandong China; ^2^ Department of Nuclear Medicine Shandong Cancer Hospital and Institute, Shandong First Medical University and Shandong Academy of Medical Science Jinan Shandong China

**Keywords:** brain metastasis, EGFR‐TKI, ICIs, uncommon mutation

## Abstract

**Background:**

Epidermal growth factor receptor tyrosine kinase inhibitors (EGFR‐TKIs) have shown significant efficacy in patients with brain metastases (BMs) from EGFR‐mutated non‐small cell lung cancer (NSCLC). However, acquired resistance is inevitable, and clinical data addressing key questions across treatment stages remain insufficient, limiting the formulation of precise treatment strategies.

**Methods:**

This retrospective study analyzed 302 EGFR‐mutant NSCLC patients with BMs treated at Shandong Cancer Hospital (2014–2022). Patients were divided into three cohorts: cohort A (first‐/second‐generation EGFR‐TKIs without third‐generation use), cohort B (first‐/second‐generation followed by third‐generation EGFR‐TKIs), and cohort C (first‐line third‐generation EGFR‐TKIs). Survival outcomes were evaluated using Kaplan–Meier and Cox regression analyses across three treatment stages. Propensity score matching (PSM) adjusted for baseline imbalances.

**Results:**

Third‐generation EGFR‐TKIs demonstrated superior progression‐free survival (PFS) in first‐line therapy compared to earlier‐generation agents (median PFS1: 14.2 vs. 11.2 months; *p* = 0.0021), particularly for intracranial control (median iPFS1: 18.0 vs. 12.2 months; *p* = 0.0058). Patients with uncommon EGFR mutations had significantly shorter PFS on third‐generation EGFR‐TKIs than those with common mutations (4.4 vs. 12.9 months; *p* = 0.012). After resistance, combination therapy with immune checkpoint inhibitors (ICIs), antiangiogenics, and chemotherapy extended overall survival (OS) versus non‐ICI regimens (median OS2: 17.3 vs. 10.4 months; *p* = 0.004).

**Conclusions:**

Third‐generation EGFR‐TKIs are effective first‐line options for BMs but show limited efficacy against uncommon mutations. Post‐resistance regimens integrating ICIs, antiangiogenics, and chemotherapy may improve survival. Reassessment of genetic and PD‐L1 status is critical for guiding sequential therapy.

## Graphical abstract

Explore the issues of interest at different stages of the treatment process for patients with EGFR‐mutated non‐small cell lung cancer with brain metastasis during the first‐line EGFR‐TKIs treatment and the subsequent therapy after the failure of EGFR‐TKIs.

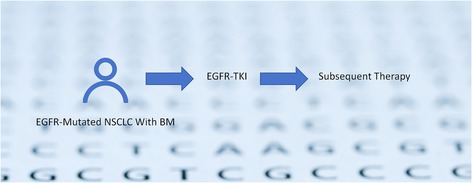

**Abbreviations**
An: antiangiogenic therapy
ARM: samplification refractory mutation system
Ch: chemotherapy
CI: confidence interval
CR: complete response
EG: EGFR‐TKIs
EGFR: TKIepidermal growth factor receptor tyrosine kinase inhibitor
ICIs: immune checkpoint inhibitors
iCR: intracranial complete response
Im: immunotherapy
iORR: intracranial objective response rate
iPD: intracranial progressive disease
iPR: intracranial partial response
iSD: intracranial stable disease
KPS: Karnofsky Performance Status
NGS: next‐generation sequencing
NSCLC: non‐small‐cell lung cancer
ORR: objective response rate
OS: overall survival
PD: progressive disease
PFS: progression‐free survival
PR: partial response
PSM: propensity score matching
Ra: radiotherapy
SD: stable disease

## 1 Introduction

Brain metastasis (BM) is a frequent and devastating complication of advanced non‐small cell lung cancer (NSCLC), occurring in up to 26% of patients and significantly worsening prognosis and quality of life [1, 2]. Epidermal growth factor receptor tyrosine kinase inhibitors (EGFR‐TKIs) have revolutionized the management of EGFR‐mutant NSCLC by improving survival and delaying disease progression [3, 4]. Third‐generation EGFR‐TKIs, such as osimertinib, offer enhanced blood–brain barrier penetration and efficacy against both activating EGFR mutations (e.g., exon 19 deletions and 21 L858R) and the T790M resistance mutation, establishing them as a first‐line standard for EGFR‐mutant NSCLC with BMs [5, 6].

Despite these advances, acquired resistance to third‐generation EGFR‐TKIs remains inevitable, with most patients progressing within 13–18 months [7, 8]. Subsequent therapeutic strategies after resistance—including continued targeted therapy, chemotherapy, antiangiogenic agents, immune checkpoint inhibitors (ICIs), and radiotherapy—are heterogeneous, and evidence guiding optimal combinations is limited [9, 10, 11]. Notably, the efficacy of third‐generation EGFR‐TKIs in patients with uncommon EGFR mutations (e.g., exon 20 insertions, G719X) remains understudied, as clinical trials often exclude these populations [12]. Furthermore, while ICIs have transformed NSCLC treatment, their role in EGFR‐mutant NSCLC post‐TKI resistance is controversial, with conflicting data on the impact of PD‐L1 expression and T790M status [13].

Current studies predominantly focus on initial responses to EGFR‐TKIs, leaving critical gaps in understanding outcomes across sequential treatment stages. Specifically, there is a paucity of real‐world data comparing the efficacy of third‐generation EGFR‐TKIs as first‐line therapy versus their use after progression on earlier‐generation agents. Additionally, the prognostic implications of combining ICIs with antiangiogenic therapy and chemotherapy post‐TKI resistance remain poorly characterized. Therefore, we conducted a comprehensive three‐stage, three‐cohort retrospective study to explore the survival outcomes of EGFR‐mutant NSCLC patients with BMs using Kaplan–Meier and Cox regression analyses. This study aims to bridge existing knowledge gaps, facilitate evidence‐based decision‐making, and ultimately improve the overall management and prognosis of this challenging patient population.

## 2 Materials and Methods

### 2.1 Patients

EGFR‐mutated NSCLC patients with BMs who were first diagnosed in Shandong Cancer Hospital from January 1, 2014 to December 31, 2022 were retrospectively collected. The institutional review board and ethics committee of Shandong Cancer Hospital approved the study. And because it was a single‐center retrospective study, patient informed consent was waived. Eligibility criteria were as follows: (1) NSCLC was confirmed by pathological examination after tissue obtained by fibrobronchoscopy or lung puncture; (2) at least one visible brain metastasis on magnetic resonance imaging (MRI) before treatment; (3) EGFR mutation was confirmed by amplification refractory mutation system (ARMS) or next‐generation sequencing (NGS); (4) received EGFR‐TKIs treatment, including gefitinib, erlotinib, icotinib, afatinib, osimertinib, almonertinib, or furmonertinib. Exclusion criteria were as follows: (1) BMs with extensive meningeal metastasis; (2) poor quality of MRI prevented accurate identification of BMs; (3) EGFR‐TKIs were used for non‐first‐line treatment; (4) incomplete medical records or detailed information of patients could not be obtained through telephone follow‐up; (5) concurrent local therapy with first‐line EGFR‐TKI treatment. Baseline characteristics of patients considered in this study included age, gender, smoking history, location of primary tumor, TNM staging [14], combined distant metastasis except brain, Karnofsky Performance Status (KPS), number of BMs, symptoms of BMs, and EGFR mutation type. Distant metastases other than brain metastases included liver metastases, bone metastases, adrenal gland metastases, pancreatic metastases, and distant lymph node metastases. Among the mutations, exon 19 deletion (*n* = 132, 43.7%), 21 L858R (*n* = 154, 61.0%), and T790M (*n* = 81, 26.8%) were considered as common mutations, while exon 20 insertion (*n* = 2, 0.7%), G719X (*n* = 6, 2.0%), L861Q (*n* = 3, 1.0%), S768I (*n* = 4, 1.3%), and the rest (*n* = 5, 1.7%) were considered as uncommon mutations [15]. Time to be collected included initial application of EGFR‐TKIs, EGFR‐TKIs resistance, subsequent therapy, relapse after drug change, and death. For patients treated with third‐generation EGFR‐TKIs after progression of low‐level EGFR‐TKIs, the time of first application of third‐generation EGFR‐TKIs were also collected. Patients were divided into three cohorts: cohort A was patients who received first‐line first‐ or second‐generation EGFR‐TKIs and did not receive third‐generation EGFR‐TKIs after resistance; cohort B was patients who received first‐ or second‐generation EGFR‐TKIs and received third‐generation EGFR‐TKIs after resistance; and cohort C was patients who received third‐generation EGFR‐TKIs as first‐line treatment.

### 2.2 Follow‐Up and End Points

After the initial EGFR‐TKIs treatment, patients underwent chest computed tomography (CT) and brain MRI every 1–3 months, and the detailed information of chest CT and brain MRI was obtained through the medical record system. For patients with incomplete medical records, telephone follow‐up was conducted to obtain specific information. Systemic efficacy was evaluated according to RECIST 1.1 criteria [16], and intracranial efficacy was evaluated according to RANO criteria [17]. The definition of good responses encompassed complete response (CR) and partial response (PR), while poor responses were characterized by stable disease (SD) and progressive disease (PD). Progression‐free survival 1 (PFS1) was the time from the start of first‐line EGFR‐TKIs treatment to the first progression or death in all patients, and intracranial PFS1 (iPFS1) was the time to intracranial progression or death. Objective response rate 1 (ORR1) was the proportion of patients achieving PR and CR on first‐line EGFR‐TKIs treatment and iORR1 was the proportion of patients achieving iPR and iCR on first‐line EGFR‐TKIs treatment. PFS2 was the time from the start of switching to third‐generation EGFR‐TKIs treatment to recurrent progression or death in cohort B, and iPFS2 was the time to recurrent intracranial progression or death. ORR2 was the proportion of patients achieving PR and CR after switching to third‐generation EGFR‐TKIs treatment and iORR2 was the proportion of patients achieving iPR and iCR. PFS3 was the time from the start of subsequent treatment after EGFR‐TKIs resistance to recurrent progression. ORR3 was the proportion of subsequent treatment after switching to EGFR‐TKIs resistance to PR + CR. OS1 and OS2 represent the time from the start of first‐line EGFR‐TKIs treatment and from the start of subsequent therapy to death from any cause, respectively.

### 2.3 Three Stages and Key Clinical Questions

**FIGURE 1:**
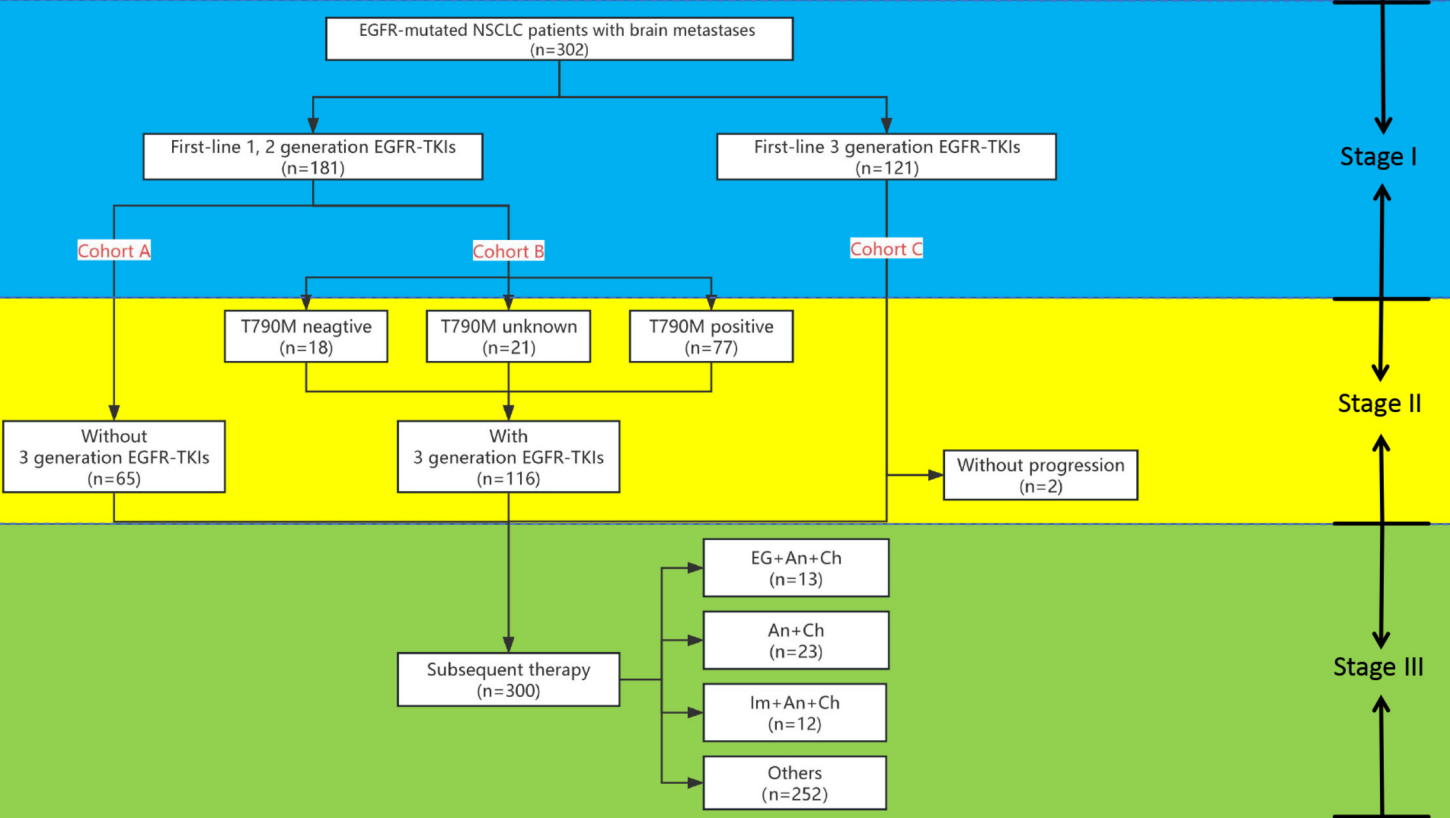
General process of this study.

This study was divided into three stages, each of which addressed different key clinical questions. The overall flow was shown in Figure 1. Stage 1 started from the first‐line EGFR‐TKIs treatment of all patients to the first progression or death. The key questions were the differences in PFS1 and short‐term systemic efficacy between first‐line low‐level EGFR‐TKIs (cohorts A + B) and first‐line third‐generation EGFR‐TKIs (cohort C). In addition, the differences in iPFS1 and short‐term intracranial efficacy between first‐line low‐level EGFR‐TKIs (cohort A) and first‐line third‐generation EGFR‐TKIs (cohort C) were also considered.

Stage 2 began with a switch to third‐generation EGFR‐TKIs after failure of first‐line low‐level EGFR‐TKIs and continued until further progression or death. Firstly, we compared the differences in PFS2, iPFS2, and response between the third‐generation EGFR‐TKIs used as first‐line therapy (cohort C, stage 1) and as subsequent therapy after progression of low‐level drugs (cohort B, stage 2). Secondly, we also compared the differences between different T790M mutations. Finally, we analyzed whether there was an association between the PFS1 of first‐line use of low‐level EGFR‐TKIs and the subsequent PFS2 of switching to third‐generation agents in cohort B.

Stage 3 began when the three cohorts received further treatment after each EGFR‐TKIs progression, until further progression or death, with cohort B experiencing twice progressions. Subsequent therapies included continued EGFR‐TKIs, chemotherapy, antiangiogenic therapy, ICIs, and local radiotherapy. The key questions were PFS3 and OS2 of subsequent various therapies alone or in combination. We focused on comparing the efficacy of three systemic combination therapies: EGFR‐TKIs, antiangiogenic therapy and chemotherapy (EG + An + Ch), antiangiogenic therapy and chemotherapy (An + Ch), and ICIs, antiangiogenic therapy and chemotherapy (Im + An + Ch). Additionally, we compared the differences in OS1 among the three cohorts and evaluated the disparities in PFS3 and OS2 between patients with positive and negative T790M mutations who received ICIs treatment.

### 2.4 Statistical Analysis

Pearson chi‐square and Fisher's precision test were used to determine differences in baseline characteristics between different groups, and for those with significant between‐group differences, propensity score matching (PSM) [18] was first used to eliminate the effect. Kaplan–Meier method and log‐rank test were used for PFS and OS analysis, and the median PFS, OS, and corresponding 95% confidence intervals (95% CI) were recorded, as well as the between‐group differences when comparing multiple groups. Cox proportional hazards regression analysis was used to screen for independent risk factors and calculate HRs and 95% CIs. Logistic regression was used to determine the association between systemic and intracranial efficacy with low‐level EGFR‐TKIs and subsequent switch to third‐generation EGFR‐TKIs. The statistical significance was determined when the P value was less than 0.05. The statistical analyses were conducted using SPSS (version 26.0), including Pearson chi‐square test, Fisher's precision test, Cox proportional hazards regression analysis, and logistic regression analysis. Additionally, Kaplan–Meier analysis, log‐rank test and bar chart were performed on R (version 4.2.2) with the utilization of survival and surviviner software packages.

## 3 Results

### 3.1 Patients

A total of 302 patients were enrolled in the study, of whom 65 (21.5%) were enrolled in cohort A, 116 (38.4%) in cohort B, and 121 (40.1%) in cohort C. The median follow‐up time for all patients was 28.5 months (17.2–74.5, range), with a median PFS1 of 12.3 months (11.6–13.3, 95% CI), and a median OS1 of 29.6 months (27.5–31.7, 95% CI). The follow‐up period concluded on January 14, 2024. In cohort A, all patients died. In cohort B, all patients experienced twice resistance to EGFR‐TKIs, of whom 114 experienced a third progression during subsequent therapy, and 105 died after progression. In cohort C, 119 patients experienced resistance to third‐generation EGFR‐TKIs, of whom 115 again experienced progression during subsequent therapy, and 87 died after progression. Baseline characteristics of the three groups were presented in Table 1.

**TABLE 1:** Baseline characteristics of patients in three cohorts when initially receiving EGFR‐TKI treatment.

Baseline characteristic	Cohort			*p* value
	B (*n* = 116)	C (*n* = 121)	A (*n* = 65)	
Age				0.821
< 65	67 (57.8%)	65 (53.7%)	36 (55.4%)	
≥ 65	49 (42.2%)	56 (46.3%)	29 (44.6%)	
Gender				0.729
Male	45 (38.8%)	48 (65.8%)	29 (44.6%)	
Female	71 (61.2%)	73 (34.2%)	36 (55.4%)	
Smoking history				0.545
No	93 (80.2%)	101 (83.5%)	50 (76.9%)	
Yes	23 (19.8%)	20 (16.5%)	15 (23.1%)	
Location				0.247
Right lung	64 (55.2%)	69 (57.0%)	29 (44.6%)	
Left lung	52 (44.8%)	52 (43.0%)	36 (55.4%)	
T stage				0.806
< 2	30 (25.9%)	30 (24.8%)	14 (21.5%)	
≥ 2	86 (74.1%)	91 (75.2%)	51 (78.5%)	
N stage				0.639
< 3	70 (60.3%)	66 (54.5%)	36 (55.4%)	
≥ 3	46 (39.7%)	55 (45.5%)	29 (44.6%)	
Other metastasis				0.488
No	44 (37.9%)	38 (31.4%)	25 (38.5%)	
Yes	72 (62.1%)	83 (68.6%)	40 (61.5%)	
KPS				0.117
< 80	78 (67.2%)	75 (62.0%)	50 (76.9%)	
≥ 80	38 (32.8%)	46 (38.0%)	15 (23.1%)	
BM number				0.386
< 5	60 (51.7%)	71 (58.7%)	32 (49.2%)	
≥ 5	56 (48.3%)	50 (41.3%)	33 (50.8%)	
BM symptom				0.824
No	98 (84.5%)	99 (81.8%)	55 (84.6%)	
Yes	18 (15.5%)	22 (18.2%)	10 (15.4%)	
EGFR mutation				0.219
Common	108 (93.1%)	116 (95.9%)	58 (89.2%)	
Uncommon	8 (6.9%)	5 (4.1%)	7 (10.8%)	

Abbreviations: BM, brain metastases; KPS, Karnofsky performance status.

### 3.2 Stage 1

**FIGURE 2:**
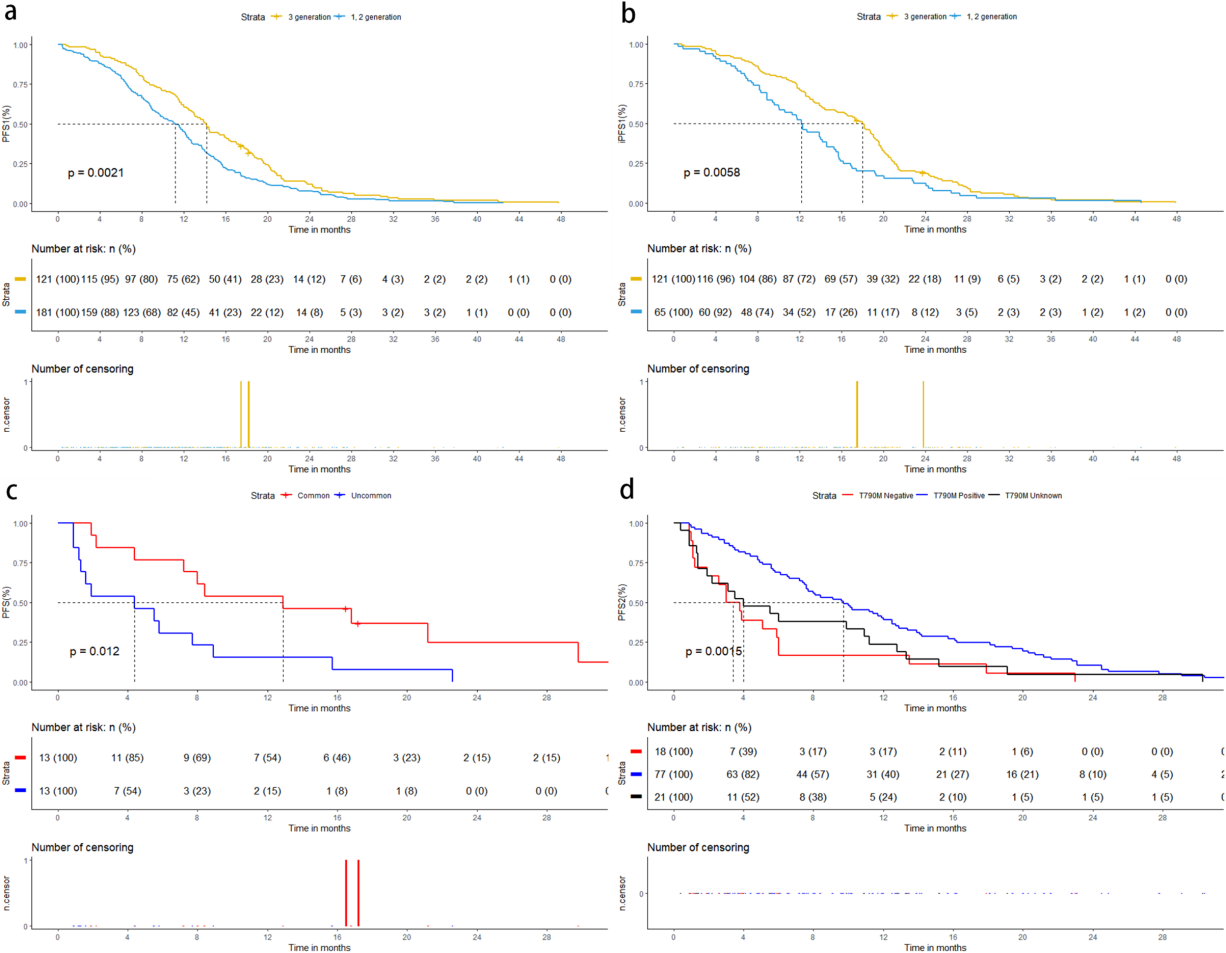
Kaplan–Meier curves for (a) PFS1 and (b) iPFS1 of first−/second‐generation vs. third‐generation EGFR‐TKIs in first‐line treatment; (c) PFS for common and uncommon mutations; (d) PFS2 by T790M status (positive, negative, unknown).

Univariate and multivariate Cox proportional hazards regression analysis **(**Table 2) showed that low‐level agents used in first‐line therapy, lower KPS (< 80), and more brain metastases (≥ 5) were independent risk factors for PFS1. Further comparison of efficacy between first‐line therapy with first‐ or second‐generation EGFR‐TKIs and first‐line therapy with third‐generation EGFR‐TKIs showed no significant differences in baseline characteristics between cohort (A + B) and cohort C (Table S1). The median PFS1 of cohort (A + B) was 11.2 months (9.6–12.3, 95% CI), ORR1 was 77.3% (69.8%–84.8%, 95% CI), and iORR1 was 80.6% (73.6%–87.6%, 95% CI). The median PFS1 of cohort C was 14.2 months (12.7–16.3, 95% CI), ORR1 was 80.9% (73.9%‑87.9, 95% CI), and iORR1 was 81.8% (74.9%–88.7%, 95% CI). Kaplan–Meier survival analysis (Figure 2a) showed that PFS1 was longer with first‐line third‐generation EGFR‐TKIs than with low‐level EGFR‐TKIs (*p* = 0.0021). Chi‐square test showed no significant differences in short‐term systemic efficacy (*p* = 0.476) and intracranial efficacy (*p* = 0.881). Further comparison of iPFS1 (Figure 2b) between first‐line low‐level EGFR‐TKIs (cohort A) and first‐line third‐generation EGFR‐TKIs (cohort C) showed that the median iPFS1 of cohort C was 18.0 months (15.4–19.3, 95% CI), and the median iPFS1 of cohort A was 12.2 months, demonstrating that iPFS of first‐line third‐generation EGFR‐TKIs was superior to first‐line low‐level agents (*p* = 0.0058). To compare the effect of mutation types on PFS with third‐generation EGFR‐TKIs, we performed a PSM between EGFR uncommon mutations and common mutations (Table 3). Then, Kaplan–Meier survival analysis (Figure 2c) showed that the PFS of common mutations and uncommon mutations were 12.9 months (7.2‐NA, 95% CI) and 4.4 months (1.3‐NA, 95% CI), respectively, with a significant difference (*p* = 0.012).

**TABLE 2:** Univariable and multivariable cox proportional hazard regression analysis of risk factors potentially associated with PFS1.

Clinical characteristics	Univariable analysis *p* value	Multivariable analysis			
		*p* value	Exp(B)	95% CI	
First‐line treatment (3 generation vs. 1, 2 generation)	**0.002**	**0.012**	1.350	1.068	1.708
Age (< 65 vs. ≥ 65)	0.809				
Gender (male vs. female)	**0.046**	0.530	0.916	0.698	1.204
Smoking history (no vs. yes)	**0.014**	0.088	1.343	0.957	1.884
Location (left lung vs. right lung)	0.490				
T stage (< 2 vs. ≥ 2)	0.435				
N stage (< 3 vs. ≥ 3)	0.440				
Other metastasis (no vs. yes)	0.439				
KPS (< 80 vs. ≥ 80)	**0.008**	**0.008**	0.714	0.558	0.914
BM number (< 5 vs. ≥ 5)	**0.000**	**0.000**	1.762	1.388	2.236
BM symptom (no vs. yes)	0.263				
EGFR mutation (common vs. uncommon)	0.188				

*Note:* Bolded numbers represent statistically significant data points (*p* < 0.05) that support the study's conclusions.

Abbreviations: BM, brain metastases; CI, confidence interval; Exp (B), odds ratio.

**TABLE 3:** The PSM of patients with common and uncommon mutations receiving third‐generation EGFR‐TKIs treatment.

Baseline characteristic	Before PSM		*p* value	After PSM		*p* value
	Common mutation (*n* = 224)	Uncommon mutation (*n* = 13)		Common mutation (*n* = 224)	Uncommon mutation (*n* = 13)	
Cohort (B/C)	108/116	8/5	0.402	7/6	8/5	1.000
Age (< 65/≥ 65)	125/99	7/6	1.000	9/4	7/6	0.688
Gender (male/female)	85/139	8/5	0.141	8/5	8/5	1.000
Smoking history (no/yes)	183/41	11/2	1.000	8/5	11/2	0.378
Location (left lung/right lung)	125/99	8/5	0.779	7/6	8/5	1.000
T stage (< 2/≥ 2)	58/166	2/11	0.525	5/8	2/11	0.378
N stage (< 3/≥ 3)	127/97	9/4	0.566	5/8	9/4	0.238
Other metastasis (no/yes)	78/146	4/9	1.000	5/8	4/9	1.000
KPS (< 80/≥ 80)	145/79	8/5	0.775	8/5	8/5	1.000
BM number (< 5/≥ 5)	129/95	2/11	0.003	2/11	2/11	1.000
BM symptom (no/yes)	187/37	10/3	0.465	11/2	10/3	1.000

Abbreviations: BM, brain metastases; KPS, Karnofsky performance status.

### 3.3 Stage 2

**FIGURE 3:**
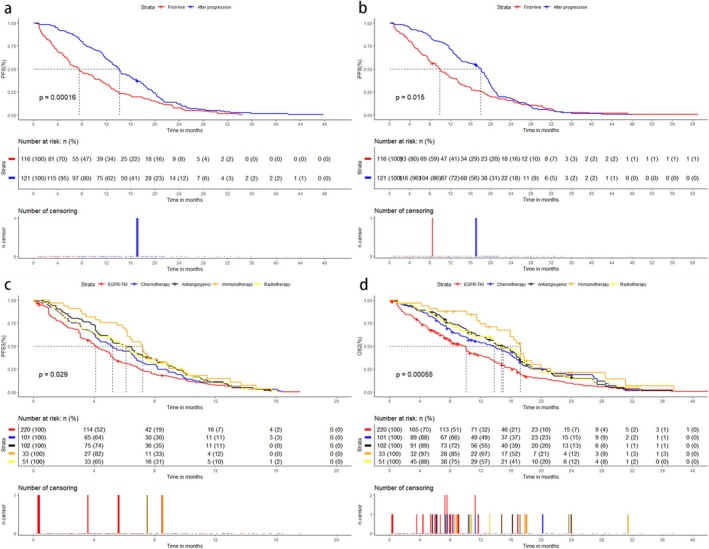
Kaplan–Meier curves for (a) PFS and (b) iPFS of third‐generation EGFR‐TKIs in first‐line vs. post‐progression settings; (c) PFS3 and (d) OS2 of subsequent therapies (EGFR‐TKIs, chemotherapy, antiangiogenics, immunotherapy, radiotherapy).

In cohort B, the median PFS2 of 116 patients was 7.6 months (5.9–10.1, 95% CI) and iPFS2 was 9.9 months (8.0–12.5, 95% CI). ORR2 was 38.8% (30.1%–47.5%, 95% CI) and iORR2 was 62.1% (53.5%–70.0%, 95% CI). And in 79 patients who switched to third‐generation agents because of intracranial progression (intracranial progression alone or both extracranial and intracranial progression), iORR2 was 43.1% (34.3%–51.9%, 95% CI). Cox proportional hazards regression analysis (Table 4) showed that negative T790M mutation, male, and EGFR uncommon mutations were independent risk factors for PFS2. Further, we compared PFS2 in patients with different T790M mutations, including 77 patients with positive, 21 patients with unknown, and 18 patients with negative. There were no significant differences in baseline characteristics among the three groups (Table S2). Kaplan–Meier survival analysis (Figure 2d) showed that the median PFS2 of positive, unknown and negative T790M mutations were 9.7 months (7.6–12.5, 95% CI), 4.0 months (1.9–12.8, 95% CI), and 3.4 months (1.9–6.1, 95% CI), respectively. PFS2 was significantly longer in T790M‐positive patients than in T790M‐negative patients (*p* = 0.0015). We also compared the PFS and iPFS of patients treated with third‐generation EGFR‐TKIs as first‐line therapy with those treated after low‐grade drug resistance, and there were no significant differences in baseline characteristics between the two groups (Table S3). Kaplan–Meier survival analysis (Figure 3a,b) showed that the PFS and iPFS of patients treated with third‐generation EGFR‐TKIs as first‐line therapy were longer than those of patients who were treated after progression of previous EGFR‐TKIs, with *p* values < 0.001 and 0.015, respectively.

**TABLE 4:** Univariable and multivariable cox proportional hazard regression analysis of risk factors potentially associated with PFS2.

Clinical characteristics	Univariable analysis *p* value	Multivariable analysis			
		*p* value	Exp(B)	95% CI	
T790M (positive vs. unknown vs. negative)	**0.002**	**0.005**			
Age (< 65 vs. ≥ 65)	0.481				
Gender (male vs. female)	**0.012**	**0.044**	0.652	0.430	0.989
Smoking history (no vs. yes)	0.231				
Location (left lung vs. right lung)	0.108				
T stage (< 2 vs. ≥ 2)	0.397				
N stage (< 3 vs. ≥ 3)	0.291				
Other metastasis (no vs. yes)	0.624				
KPS (< 80 vs. ≥ 80)	0.193				
BM number (< 5 vs. ≥ 5)	**0.029**	0.409	1.180	0.797	1.748
BM symptom (no vs. yes)	0.078				
EGFR mutation (common vs. uncommon)	**0.001**	**0.001**	3.478	1.612	7.505

*Note:* Bolded numbers represent statistically significant data points (*p* < 0.05) that support the study's conclusions.

Abbreviations: BM, brain metastases; CI, confidence interval; Exp (B), odds ratio.

### 3.4 Stage 3

**FIGURE 4:**
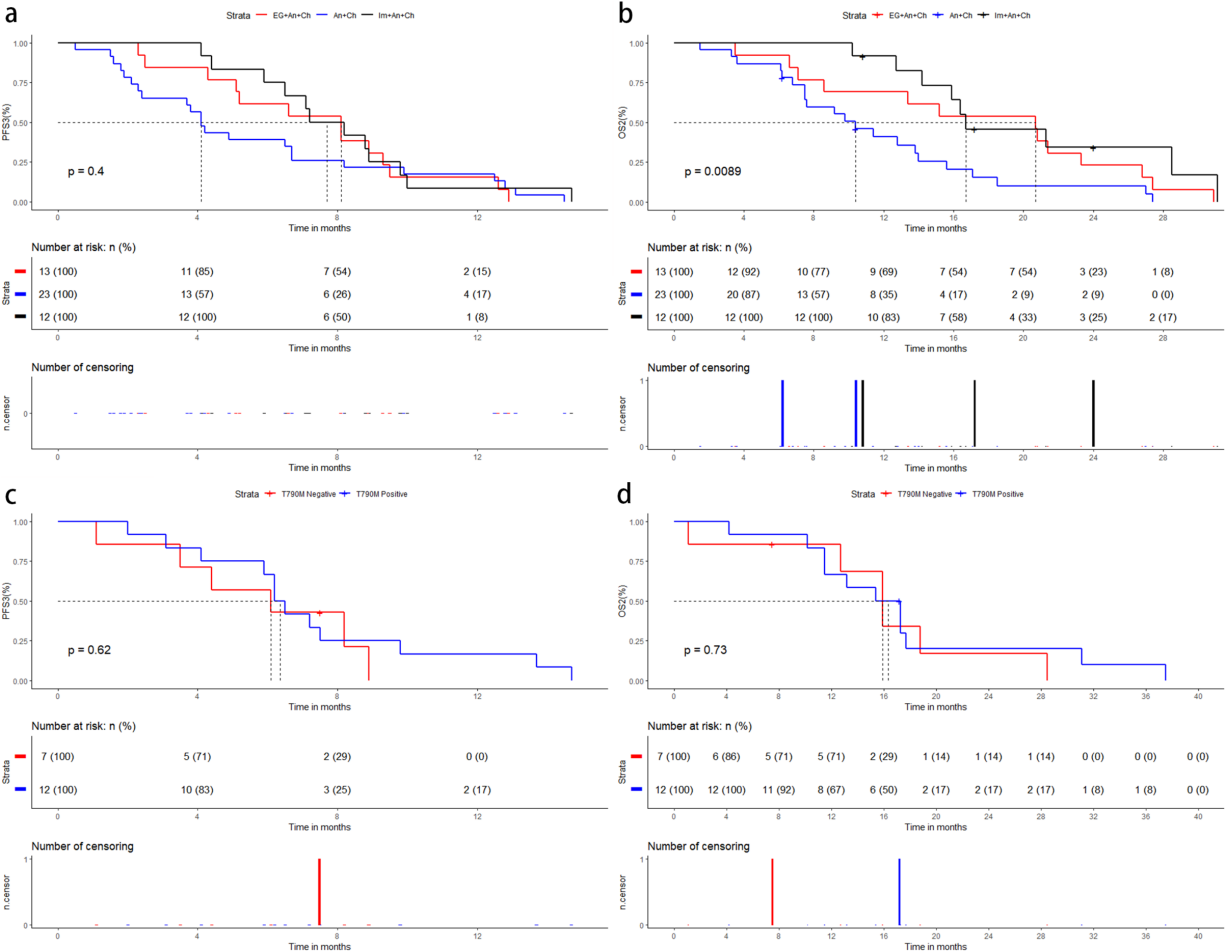
Kaplan–Meier curves for (a) PFS3 and (b) OS2 with Im + An + Ch, An + Ch, and EG + An + Ch; (c) PFS3 and (d) OS2 by T790M status (positive/negative) with immunotherapy. EG, EGFR‐TKIs; Ch, chemotherapy; An, antiangiogenic therapy; Im, immunotherapy.

**FIGURE 5:**
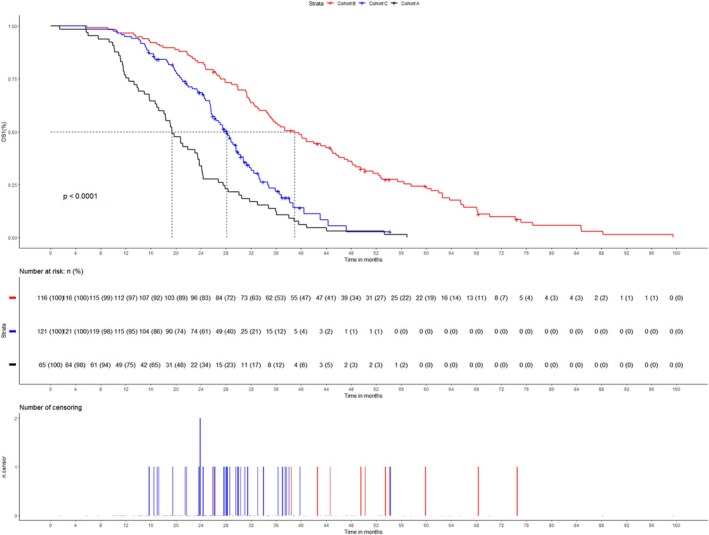
Kaplan–Meier curves of OS1 for cohort A, B, and C.

Subsequently, 300 of 302 patients developed resistance to EGFR‐TKIs. Treatment modalities after progression included continued EGFR‐TKIs, chemotherapy, antiangiogenic therapy, ICIs, and local radiotherapy. The first efficacy evaluation was performed in 297 patients who received subsequent treatment, and the overall ORR3 was 15.5% (9.1–21.9, 95% CI). The specific subsequent treatment modalities, along with the efficacy and prognosis of each modality, were shown in Table 5. Cox proportional hazards regression analysis revealed that advanced age, presence of other distant metastases, and nonuse of antiangiogenic agents were identified as independent risk factors for PFS3. Additionally, higher T stage, presence of other distant metastases, and a subsequent treatment regimen excluding chemotherapy, antiangiogenic therapy, ICI or radiotherapy were determined to be independent risk factors for OS2 (Tables S4 and S5). However, there was no significant difference in PFS3 among the cohorts (*p* = 0.757). Kaplan–Meier curves for subsequent treatment with EGFR‐TKIs, chemotherapy, antiangiogenic agents, ICIs, and local radiotherapy showed median PFS3 of 4.1 months (3.7–5.0, 95% CI), 5.2 months (4.2–6.7, 95% CI), 6.1 months (5.1–7.2, 95% CI), 7.2 months (6.2–8.9, 95% CI), and 6.1 months (4.2–7.6, 95% CI) (Figure 3c), and median OS2 of 10.1 months (8.0–10.5, 95% CI), 13.8 months (10.2–16.2, 95% CI), 15.0 months (12.7–16.8 95% CI), 17.3 months (15.9–20.0, 95% CI), and 14.8 months (11.0–17.7, 95% CI) (Figure 3d). We also compared PFS3 and OS2 among three systemic combinations, including EGFR‐TKIs + antiangiogenic therapy + chemotherapy (EG + An + Ch), antiangiogenic therapy + chemotherapy (An + Ch), and immunotherapy + antiangiogenic therapy + chemotherapy (Im + An + Ch). Baseline characteristics of patients in the three combinations were not significantly different (Table S6). Kaplan–Meier survival analysis (Figure 4a,b) showed no significant difference in PFS3 among the three groups (*p* = 0.4), but a significant difference in OS2 (*p* = 0.0089). The comparison between the three groups showed that the Im + An + Ch group had a longer OS2 than the An + Ch group (*p* = 0.004), but no significant difference compared with the EG + An + Ch group (*p* = 0.071). The median PFS3 and OS2 in the Im + An + Ch group were 7.2 months (6.2–8.9, 95% CI) and 17.3 months (15.9–20.0, 95% CI), respectively. Baseline characteristics of T790M‐positive and T790M‐negative patients treated with ICIs did not show significant differences (Table S7). The median PFS3 were respectively 6.4 months and 6.1 months, and the median OS2 were respectively 16.4 months and 15.9 months, without statistically significant disparities (Figure 4c,d). Finally, Cox proportional hazards regression analysis (Table S8) showed that first‐line non‐third‐generation EGFR‐TKIs, male, lower KPS, and higher number of BMs were independent risk factors for OS1. Kaplan–Meier analysis (Figure 5) showed that OS1 of cohort B was significantly longer than cohort C and cohort A (*p* < 0.0001), with median OS1 of 38.9 months (34.9–45.1, 95% CI), 28.1 months (25.7–30.3, 95% CI), and 19.4 months (17.2–23.7, 95% CI), respectively.

**TABLE 5:** Detailed subsequent therapy and efficacy after progression of EGFR‐TKI therapy.

Subsequent therapy	Patients(*N* = 300)	ORR3	DCR3	mPFS3 (month)	95% CI		WP (*N* = 6)	OS2	95% CI		WD (*N* = 43)
EG	124	2.4%	50.7%	3.1	3.0	3.8	4	6.1	5.0	8.3	21
EG + An	30	20.0%	70.0%	5.1	4.0	7.7	0	11.5	9.3	16.6	3
EG + An + Ch	13	23.1%	84.6%	8.1	5.1	NA	0	20.7	8.6	NA	0
EG + Ch	20	10.0%	65.0%	5.1	4.0	8.2	0	10.4	6.7	29.0	2
An + Ch	23	17.3%	69.4%	4.1	2.4	8.2	0	10.4	7.5	15.6	2
Ra + EG	21	19.0%	76.1%	6.4	3.6	11.8	0	14.1	8.3	23.0	3
Ra + EG + An	12	16.6%	83.3%	5.7	4.2	NA	0	16.7	13.8	NA	2
Ra + EG + An + Ch	1	100.0%	100.0%	3.0	NA	NA	0	9.4	NA	NA	0
Ra + EG + Ch	1	0.0%	100.0%	6.8	NA	NA	0	9.2	NA	NA	0
Ra + An + Ch	6	16.6%	83.2%	9.7	5.2	NA	0	22.3	11.0	NA	0
Ra + Ch	4	0.0%	25.0%	1.7	1.4	NA	0	5.8	5.2	NA	0
Ra + Im + An	3	33.3%	100.0%	7.6	6.1	NA	0	18.1	17.3	NA	1
Ra + Im + Ch	3	66.7%	100.0%	6.4	2.0	NA	0	20.0	17.3	NA	1
Ch	11	18.1%	45.5%	3.1	2.0	NA	0	8.1	5.1	NA	2
Im	2	0.0%	100.0%	6.2	NA	NA	0	11.5	NA	NA	0
Im + EG + An	1	0.0%	100.0%	6.0	NA	NA	0	22.3	NA	NA	0
Im + An	6	66.7%	100.0%	11.5	7.2	NA	2	37.5	17.8	NA	3
Im + An + Ch	12	66.7%	100.0%	7.7	6.5	NA	0	16.7	15.9	NA	3
Im + Ch	7	42.8%	71.5%	3.5	2.3	NA	0	11.9	4.2	NA	0

Abbreviations: An, antiangiogenic therapy; Ch, chemotherapy; EG, EGFR‐TKI; Im, immunotherapy; Ra, radiotherapy; WD, number of patients without death; WP, number of patients without disease progression.

## 4 Discussion

Extensive clinical trials have confirmed that the third‐generation EGFR‐TKIs can better control BMs than traditional EGFR‐TKIs [19, 20, 21]. In this retrospective study, we included a larger number of EGFR‐mutated NSCLC patients with BMs who received first‐line EGFR‐TKIs treatment. We also explored key clinical questions at different stages of therapy, which not only enriched the clinical data for this patient population but also further confirmed the significant role of EGFR‐TKIs. However, in a phase II clinical trial [22], the median PFS of patients with uncommon mutations and BMs treated with osimertinib was only 5.4 months. Our study also confirmed that third‐generation EGFR‐TKIs exhibit superior efficacy in treating common mutations (19del, 21 L858R, and T790M) compared to uncommon mutations (other mutation types). Notably, the median PFS1 of the nine patients with uncommon mutations who received afatinib as first‐line therapy was 9.0 months (95% CI: 5.2–12.8), with an ORR1 of 88.9%. Additionally, one patient with an uncommon mutation, who initially received a third‐generation EGFR‐TKI as first‐line therapy and later switched to afatinib due to disease progression, was re‐evaluated as SD, with a PFS3 of 11.5 months. In contrast, four patients with uncommon mutations who used afatinib as first‐line treatment were all assessed as PD after switching to third‐generation EGFR‐TKIs upon disease progression. These findings suggest that certain types of first‐ or second‐generation EGFR‐TKIs may have better efficacy than third‐generation drugs in treating uncommon mutations, indicating that third‐generation drugs cannot entirely replace previous generations of medications.

Although no differences in efficacy were observed among different cohorts in our study when receiving subsequent treatment, extensive prospective studies consistently demonstrate that first‐line third‐generation EGFR‐TKIs treatment can lead to fewer adverse events (AE), thereby enabling patients to maintain a better overall condition for further treatment even after disease progression, ultimately resulting in an improved prognosis [23, 24]. Continuation of EGFR‐TKIs after disease progression encompasses maintaining the same drug at the same dosage, escalating the dosage of the same drug, transitioning to an alternative medication, and combining two targeted therapies. Continuing the original regimen after disease progression is generally associated with suboptimal efficacy; however, dose escalation may potentially overcome resistance and improve clinical outcomes, provided that the drug is well tolerated by the patient [25]. Goldstein et al. [26] demonstrated that patients who experienced isolated intracranial progression following osimertinib 80 mg treatment could still benefit from a dose escalation to 160 mg. Ding et al. [27] reported three cases where patients switched to almonertinib after failing osimertinib, resulting in a PR and a median PFS of 10 months upon switching. We also observed one patient achieving a PR after transitioning to almonertinib due to resistance against osimertinib, with a PFS of 6.8 months. Furthermore, osimertinib may lead to loss of T790M mutation and tertiary mutations; hence switching to lower‐grade EGFR‐TKIs remains effective [28]. For patients with MET amplification or overexpression after osimertinib resistance, multiple clinical trials have also confirmed that double‐target therapy can play a better role [29, 30, 31].

Previous studies had demonstrated the synergistic effect of multi‐target antiangiogenic therapy in combination with EGFR‐TKIs [32]. Furthermore, the efficacy of combining anti‐angiogenic drugs with ICIs or chemotherapy remained effective for subsequent treatment in patients with EGFR‐TKIs resistance [33, 34, 35]. Our study further confirmed that Im + An + Ch exhibited a superior OS2 compared to An + Ch, while no significant difference was observed between EG + An + Ch and Im + An + Ch. The effectiveness of ICIs was associated with PD‐L1 expression levels, and previous studies had also indicated that T790M negativity led to higher PD‐L1 expression, resulting in improved efficacy of ICIs treatment [36, 37]. However, this observation was not replicated in our study possibly due to the limited number of eligible patients. Reassessing PD‐L1 expression after developing resistance to EGFR‐TKIs could guide subsequent treatment. Further investigation was required to determine whether switching to different types of EGFR‐TKIs combined with antiangiogenic drugs and chemotherapy is more effective for patients with low PD‐L1 expression levels. Additionally, the combination of local radiotherapy was also an effective treatment modality for improving the prognosis of patients with oligometastasis or oligoprogression [38, 39, 40].

We also observed that the OS1 of cohort B exceeded that of the other two groups. On one hand, there were still 30 patients in cohort C who had not yet reached an outcome, and considering the presenting clinical trials on third‐generation EGFR‐TKIs, it was possible for the OS1 of cohort C to further improve over time [41]. On the other hand, the third‐generation EGFR‐TKIs could effectively target tumor cells and control gene mutations even after first‐line treatment failure with low‐level EGFR‐TKIs. Therefore, the emergence of new targeted drugs that could overcome the resistance of third‐generation drugs was also needed.

There were also limitations in our study. Firstly, it should be noted that this was a retrospective study conducted at a single center, and the sample size might be slightly inadequate for addressing certain research issues of interest. In future investigations, it would be beneficial to expand the sample size further and conduct multi‐center clinical trials in order to validate our findings. Secondly, the efficacy of different drug types within the same class was not separately analyzed, which may have impacted our results.

## 5 Conclusions

In conclusion, our study reaffirmed the significant advantage of EGFR‐TKIs in EGFR‐mutated NSCLC patients with BMs. However, the efficacy was slightly inferior in patients with uncommon EGFR mutations. Following drug resistance, gene testing and PD‐L1 measurement were necessary to determine an individualized subsequent treatment plan that closely correlates with prognosis. Combining ICIs, antiangiogenic therapy, and chemotherapy may be more beneficial than not combining ICIs. Future development of new generation EGFR‐TKIs should focus on addressing drug resistance issues and better managing rare mutation types.

## Author Contributions

**Haoran Qi:** conceptualization, data curation, methodology, software, formal analysis, writing – original draft; **Qiang Qiao:** data curation, software; **Xiaorong Sun:** conceptualization, supervision; **Ligang Xing:** conceptualization, writing review and editing.

## Ethics Statement

Approval of the research protocol by an Institutional Reviewer Board: This study was carried out in accordance with the Helsinki Declaration, and the study protocol was approved by the Ethics Review Committee of Shandong Cancer Hospital in China (SDTHEC2021003100).

## Conflicts of Interest

The authors declare no conflicts of interest.

## Supporting information


**Table S1** Baseline characteristics of patients initially treated with first‐ or second‐generation EGFR‐TKIs in the first‐line (cohorts A + B), as well as those initially treated with third‐generation EGFR‐TKIs (cohort C). Abbreviations: KPS, Karnofsky Performance Status; BM, brain metastases.
**Table S2** Baseline characteristics of patients when receiving third‐generation EGFR‐TKI treatment after failing first‐line treatment with either first‐generation or second‐generation EGFR‐TKIs. Abbreviations: KPS, Karnofsky Performance Status; BM, brain metastases.
**Table S3** Baseline characteristics of patients in cohort B and cohort C when receiving treatment with third‐generation EGFR‐TKIs. Abbreviations: KPS, Karnofsky Performance Status; BM, brain metastases.
**Table S4** Univariable and multivariable Cox proportional hazard regression analysis of risk factors potentially associated with PFS3. Abbreviations: Exp (B), odds ratio; CI, confidence interval; BM, brain metastases.
**Table S5** Univariable and multivariable Cox proportional hazard regression analysis of risk factors potentially associated with OS2. Abbreviations: Exp (B), odds ratio; CI, confidence interval; BM, brain metastases.
**Table S6** Baseline characteristics of patients when receiving subsequent therapy after failure of EGFR‐TKI. Abbreviations: KPS, Karnofsky Performance Status; BM, brain metastases; EG, EGFR‐TKI; Ch, chemotherapy; An, antiangiogenic therapy; Im, immunotherapy.
**Table S7** Baseline characteristics of T790M‐positive and ‐negative patients receiving immunotherapy. Abbreviations: KPS, Karnofsky Performance Status; BM, brain metastases.
**Table S8** Univariable and multivariable Cox proportional hazard regression analysis of risk factors potentially associated with OS1. Abbreviations: Exp (B), odds ratio; CI, confidence interval; BM, brain metastases.

## Data Availability

The data that support the findings of this study are available from the corresponding author upon reasonable request.
