# Supplementary material for: Survival Outcomes in EGFR‐Mutant Non‐Small Cell Lung Cancer With Brain Metastases: Kaplan–Meier and Cox Regression Analyses Across Treatment Stages
Source: Clin Respir J. 2025 May 27;19(5):e70085. doi: 10.1111/crj.70085 (PMC12107365; doi:10.1111/crj.70085)
Supplement: Supplementary file 1 — Table S1 Baseline characteristics of patients initially treated with first‐ or second‐generation EGFR‐TKIs in the first‐line (cohorts A + B), as well as those initially treated with third‐generation EGFR‐TKIs (cohort C). Abbreviations: KPS, Karnofsky Performance Status; BM, brain metastases. Table S2 Baseline characteristics of patients when receiving third‐generation EGFR‐TKI treatment after failing first‐line treatment with either first‐generation or second‐generation EGFR‐TKIs. Abbreviations: KPS, Karnofsky Performance Status; BM, brain metastases. Table S3 Baseline characteristics of patients in cohort B and cohort C when receiving treatment with third‐generation EGFR‐TKIs. Abbreviations: KPS, Karnofsky Performance Status; BM, brain metastases. Table S4 Univariable and multivariable Cox proportional hazard regression analysis of risk factors potentially associated with PFS3. Abbreviations: Exp (B), odds ratio; CI, confidence interval; BM, brain metastases. Table S5 Univariable and multivariable Cox proportional hazard regression analysis of risk factors potentially associated with OS2. Abbreviations: Exp (B), odds ratio; CI, confidence interval; BM, brain metastases. Table S6 Baseline characteristics of patients when receiving subsequent therapy after failure of EGFR‐TKI. Abbreviations: KPS, Karnofsky Performance Status; BM, brain metastases; EG, EGFR‐TKI; Ch, chemotherapy; An, antiangiogenic therapy; Im, immunotherapy. Table S7 Baseline characteristics of T790M‐positive and ‐negative patients receiving immunotherapy. Abbreviations: KPS, Karnofsky Performance Status; BM, brain metastases. Table S8 Univariable and multivariable Cox proportional hazard regression analysis of risk factors potentially associated with OS1. Abbreviations: Exp (B), odds ratio; CI, confidence interval; BM, brain metastases. [file CRJ-19-e70085-s001.docx]

### **Table S1** Baseline characteristics of patients initially treated with first or second-generation EGFR-TKIs in the first line (cohort A+B), as well as those initially treated with third-generation EGFR-TKIs (cohort C).

| Baseline characteristic | 3 generation (Cohort C)  (n=121) | 1, 2 generation (Cohort A+B)  (n=181) | P value |
| --- | --- | --- | --- |
| Age |  |  | 0.637 |
| <65 | 65 (53.7%) | 103 (56.9%) |  |
| ≥65 | 56 (46.3%) | 78 (43.1%) |  |
| Gender |  |  | 0.905 |
| Male | 48 (39.7%) | 74 (40.9%) |  |
| Female | 73 (60.3%) | 107 (59.1%) |  |
| Smoking history |  |  | 0.373 |
| No | 101 (83.5%) | 143 (79.0%) |  |
| Yes | 20 (16.5%) | 38 (21.0%) |  |
| Location |  |  | 0.349 |
| Right lung | 69 (57.0%) | 93 (51.4%) |  |
| Left lung | 52 (43.0%) | 88 (48.6%) |  |
| T stage |  |  | 1.000 |
| <2 | 30 (24.8%) | 44 (24.3%) |  |
| ≥2 | 91 (75.2%) | 137 (75.7%) |  |
| N stage |  |  | 0.553 |
| <3 | 66 (54.5%) | 106 (58.6%) |  |
| ≥3 | 55 (45.5%) | 75 (41.4%) |  |
| Other metastasis |  |  | 0.270 |
| No | 38 (31.4%) | 69 (38.1%) |  |
| Yes | 83 (68.6%) | 112 (61.9%) |  |
| KPS |  |  | 0.133 |
| <80 | 75 (62.0%) | 128 (70.7%) |  |
| ≥80 | 46 (38.0%) | 53 (29.3%) |  |
| BM number |  |  | 0.196 |
| <5 | 71 (58.7%) | 92 (50.8%) |  |
| ≥5 | 50 (41.3%) | 89 (49.2%) |  |
| BM symptom |  |  | 0.532 |
| No | 99 (81.8%) | 153 (84.5%) |  |
| Yes | 22 (18.2%) | 28 (15.5%) |  |
| EGFR mutation |  |  | 0.237 |
| Common | 116 (95.9%) | 166 (91.7%) |  |
| Uncommon | 5 (4.1%) | 15 (8.3%) |  |

Abbreviations: KPS, Karnofsky Performance Status; BM, brain metastases.

**Table S2** Baseline characteristics of patients when receiving third-generation EGFR-TKI treatment after failing first-line treatment with either first-generation or second-generation EGFR-TKIs.

| Baseline characteristic | T790M | | | P value |
| --- | --- | --- | --- | --- |
|  | Negative(n=18) | Positive(n=77) | Unknown(n=21) |  |
| Age |  |  |  | 0.171 |
| <65 | 14 (77.8%) | 42 (54.5%) | 11 (52.4%) |  |
| ≥65 | 4 (22.2%) | 35 (45.5%) | 10 (47.6%) |  |
| Gender |  |  |  | 0.100 |
| Male | 11 (61.1%) | 26 (33.8%) | 8 (38.1%) |  |
| Female | 7 (38.9%) | 51 (66.2%) | 13 (61.9%) |  |
| Smoking history |  |  |  | 0.816 |
| No | 14 (77.8%) | 63 (81.8%) | 16 (76.2%) |  |
| Yes | 4 (22.2%) | 14 (18.2%) | 5 (23.8%) |  |
| Location |  |  |  | 0.031 |
| Right lung | 12 (66.7%) | 36 (46.8%) | 16 (76.2%) |  |
| Left lung | 6 (33.3%) | 41 (53.2%) | 5 (23.8%) |  |
| T stage |  |  |  | 0.030 |
| <2 | 1 (5.6%) | 20 (26.0%) | 9 (42.9%) |  |
| ≥2 | 17 (94.4%) | 57 (74.0%) | 12 (57.1%) |  |
| N stage |  |  |  | 0.583 |
| <3 | 10 (55.6%) | 49 (63.6%) | 11 (52.4%) |  |
| ≥3 | 8 (44.4%) | 28 (36.4%) | 10 (47.6%) |  |
| Other metastasis |  |  |  | 0.617 |
| No | 5 (27.8%) | 31 (40.3%) | 8 (38.1%) |  |
| Yes | 13 (72.2%) | 46 (59.7%) | 13 (61.9%) |  |
| KPS |  |  |  | 0.325 |
| <80 | 12 (66.7%) | 49 (63.6%) | 17 (81.0%) |  |
| ≥80 | 6 (33.3%) | 28 (36.4%) | 4 (19.0%) |  |
| BM number |  |  |  | 0.889 |
| <5 | 9 (50.0%) | 41 (53.2%) | 10 (47.6%) |  |
| ≥5 | 9 (50.0%) | 36 (46.8%) | 11 (52.4%) |  |
| BM symptom |  |  |  | 0.406 |
| No | 17 (94.4%) | 63 (81.8%) | 18 (85.7%) |  |
| Yes | 1 (5.6%) | 14 (18.2%) | 3 (14.3%) |  |
| EGFR mutation |  |  |  | 0.137 |
| Common | 15 (83.3%) | 74 (96.1%) | 19 (90.5%) |  |
| Uncommon | 3 (16.7%) | 3 (3.9%) | 2 (9.5%) |  |

Abbreviations: KPS, Karnofsky Performance Status; BM, brain metastases.

### **Table S3** Baseline characteristics of patients in cohort B and cohort C when receiving treatment with third-generation EGFR-TKIs.

| Baseline characteristic | Cohort B  (n=116) | Cohort C  (n=121) | P value |
| --- | --- | --- | --- |
| Age |  |  | 0.601 |
| <65 | 67 (57.8%) | 65 (53.7%) |  |
| ≥65 | 49 (42.2%) | 56 (46.3%) |  |
| Gender |  |  | 0.895 |
| Male | 45 (38.8%) | 48 (39.7%) |  |
| Female | 71 (61.2%) | 73 (60.3%) |  |
| Smoking history |  |  | 0.613 |
| No | 93 (80.2%) | 101 (83.5%) |  |
| Yes | 23 (19.8%) | 20 (16.5%) |  |
| Location |  |  | 0.795 |
| Right lung | 64 (55.2%) | 69 (57.0%) |  |
| Left lung | 52 (44.8%) | 52 (43.0%) |  |
| T stage |  |  | 0.282 |
| <2 | 30 (25.9%) | 24 (19.8%) |  |
| ≥2 | 86 (74.1%) | 97 (80.2%) |  |
| N stage |  |  | 0.294 |
| <3 | 70 (60.3%) | 64 (52.9%) |  |
| ≥3 | 49 (39.7%) | 47 (47.1%) |  |
| Other metastasis |  |  | 0.271 |
| No | 43 (37.1%) | 36 (29.8%) |  |
| Yes | 73 (62.9%) | 85 (70.2%) |  |
| KPS |  |  | 0.283 |
| <80 | 78 (67.2%) | 73 (60.3%) |  |
| ≥80 | 38 (32.8%) | 48 (39.7%) |  |
| BM number |  |  | 0.298 |
| <5 | 60 (51.7%) | 71 (58.7%) |  |
| ≥5 | 56 (48.3%) | 51 (41.3%) |  |
| BM symptom |  |  | 0.401 |
| No | 98 (84.5%) | 97 (80.2%) |  |
| Yes | 18 (15.5%) | 24 (19.8%) |  |
| EGFR mutation |  |  | 0.402 |
| Common | 108 (93.1%) | 116 (95.9%) |  |
| Uncommon | 8 (6.9%) | 5 (4.1%) |  |

Abbreviations: KPS, Karnofsky Performance Status; BM, brain metastases.

**Table S4** Univariable and multivariable cox proportional hazard regression analysis of risk factors potentially associated with PFS3.

| Clinical Characteristics | Univariable analysis  p value | Multivariable analysis | | | |
| --- | --- | --- | --- | --- | --- |
|  |  | P value | Exp(B) | 95% CI | |
| Cohort  (B vs.C vs. A) | 0.757 |  |  |  |  |
| Age  (<65 vs.≥65) | **0.006** | **0.047** | 1.270 | 1.003 | 1.608 |
| Gender  (Male vs.Female) | 0.887 |  |  |  |  |
| Smoking history  (No vs.Yes) | 0.269 |  |  |  |  |
| Location  (Left lung vs.Right lung) | 0.900 |  |  |  |  |
| T stage  (<2 vs.≥2) | 0.100 |  |  |  |  |
| N stage  (<3 vs.≥3) | 0.606 |  |  |  |  |
| Other metastasis  (No vs.Yes) | **0.019** | **0.039** | 1.291 | 1.013 | 1.645 |
| KPS  (<80 vs.≥80) | 0.089 |  |  |  |  |
| BM number  (<5 vs.≥5) | 0.588 |  |  |  |  |
| BM symptom  (No vs.Yes) | 0.666 |  |  |  |  |
| EGFR mutation  (Common vs.Uncommon) | 0.186 |  |  |  |  |
| *Subsequent therapy* | | | | | |
| EGFR-TKI  (Yes vs.No) | 0.135 |  |  |  |  |
| Chemotherapy  (Yes vs.No) | 0.106 |  |  |  |  |
| [Antiangiogenic therapy](https://pubmed.ncbi.nlm.nih.gov/35842983/)  (Yes vs.No) | **0.004** | **0.033** | 0.761 | 0.592 | 0.978 |
| [Immunotherapy](https://pubmed.ncbi.nlm.nih.gov/31944278/)  (Yes vs.No) | **0.017** | 0.100 | 0.725 | 0.494 | 1.064 |
| Radiotherapy  (Yes vs.No) | 0.120 |  |  |  |  |

Abbreviations: Exp(B), odds ratio; CI, confidence interval; BM, brain metastases.

**Table S5** Univariable and multivariable cox proportional hazard regression analysis of risk factors potentially associated with OS2.

| Clinical Characteristics | Univariable analysis  p value | Multivariable analysis | | | |
| --- | --- | --- | --- | --- | --- |
|  |  | P value | Exp(B) | 95% CI | |
| Cohort  (B vs.C vs. A) | 0.205 |  |  |  |  |
| Age  (<65 vs.≥65) | **0.005** | 0.076 | 1.241 | 0.978 | 1.574 |
| Gender  (Male vs.Female) | 0.324 |  |  |  |  |
| Smoking history  (No vs.Yes) | 0.760 |  |  |  |  |
| Location  (Left lung vs.Right lung) | 0.953 |  |  |  |  |
| T stage  (<2 vs.≥2) | **0.018** | **0.024** | 0.730 | 0.555 | 0.960 |
| N stage  (<3 vs.≥3) | 0.521 |  |  |  |  |
| Other metastasis  (No vs.Yes) | **0.048** | **0.014** | 1.364 | 1.064 | 1.750 |
| KPS  (<80 vs.≥80) | 0.054 |  |  |  |  |
| BM number  (<5 vs.≥5) | 0.254 |  |  |  |  |
| BM symptom  (No vs.Yes) | 0.952 |  |  |  |  |
| EGFR mutation  (Common vs.Uncommon) | 0.514 |  |  |  |  |
| *Subsequent therapy* | | | | | |
| EGFR-TKI  (Yes vs.No) | **0.023** | 0.056 | 0.706 | 0.495 | 1.008 |
| Chemotherapy  (Yes vs.No) | **0.002** | **0.004** | 0.638 | 0.469 | 0.866 |
| [Antiangiogenic therapy](https://pubmed.ncbi.nlm.nih.gov/35842983/)  (Yes vs.No) | **0.000** | **0.040** | 0.758 | 0.582 | 0.987 |
| [Immunotherapy](https://pubmed.ncbi.nlm.nih.gov/31944278/)  (Yes vs.No) | **0.003** | **0.007** | 0.551 | 0.358 | 0.848 |
| Radiotherapy  (Yes vs.No) | **0.022** | **0.006** | 0.647 | 0.474 | 0.884 |

Abbreviations: Exp(B), odds ratio; CI, confidence interval; BM, brain metastases.

**Table S6** Baseline characteristics of patients when receiving subsequent therapy after failure of EGFR-TKI.

| Baseline characteristic | Subsequent therapy | | | P value |
| --- | --- | --- | --- | --- |
|  | EG+An+Ch (n=13) | An+Ch (n=23) | Im+An+Ch (n=12) |  |
| Cohort |  |  |  | 0.053 |
| B | 3 (23.1%) | 12 (52.2%) | 6 (50.0%) |  |
| C | 4 (30.8%) | 7 (30.4%) | 6 (50.0%) |  |
| A | 6 (46.1%) | 4 (17.4%) | 0 (0.0%) |  |
| Age |  |  |  | 0.225 |
| <65 | 10 (76.9%) | 12 (52.2%) | 9 (75.0%) |  |
| ≥65 | 3 (23.1%) | 11 (47.8%) | 3 (25.0%) |  |
| Gender |  |  |  | 0.133 |
| Male | 4 (30.8%) | 14 (60.9%) | 4 (33.3%) |  |
| Female | 9 (69.2%) | 9 (39.1%) | 8 (66.7%) |  |
| Smoking history |  |  |  | 0.265 |
| No | 11 (84.6%) | 16 (69.6%) | 11 (91.7%) |  |
| Yes | 2 (15.4%) | 7 (30.4%) | 1 (8.3%) |  |
| Location |  |  |  | 0.797 |
| Right lung | 6 (46.2%) | 11 (47.8%) | 7 (58.3%) |  |
| Left lung | 7 (53.8%) | 12 (52.2%) | 5 (41.7%) |  |
| T stage |  |  |  | 0.250 |
| <2 | 1 (7.7%) | 7 (30.4%) | 2 (16.7%) |  |
| ≥2 | 12 (92.3%) | 16 (69.6%) | 10 (83.3%) |  |
| N stage |  |  |  | 0.699 |
| <3 | 7 (53.8%) | 12 (52.2%) | 8 (66.7%) |  |
| ≥3 | 6 (46.2%) | 11 (47.8%) | 4 (33.3%) |  |
| Other metastasis |  |  |  | 0.451 |
| No | 3 (23.1%) | 10 (43.5%) | 5 (41.7%) |  |
| Yes | 10 (76.9%) | 13 (56.5%) | 7 (58.3%) |  |
| KPS |  |  |  | 0.601 |
| <80 | 10 (76.9%) | 16 (69.6%) | 7 (58.3%) |  |
| ≥80 | 3 (23.1%) | 7 (30.4%) | 5 (41.7%) |  |
| BM number |  |  |  | 0.790 |
| <5 | 6 (46.2%) | 13 (56.5%) | 7 (58.3%) |  |
| ≥5 | 7 (53.8%) | 10 (43.5%) | 5 (41.7%) |  |
| BM symptom |  |  |  | 0.806 |
| No | 11 (84.6%) | 19 (82.6%) | 9 (75.0%) |  |
| Yes | 2 (15.4%) | 4 (17.4%) | 3 (25.0%) |  |
| EGFR mutation |  |  |  | 0.199 |
| Common | 13 (100.0%) | 22 (95.7%) | 10 (83.3%) |  |
| Uncommon | 0 (0.0%) | 1 (4.3%) | 2 (16.7%) |  |

Abbreviations: KPS, Karnofsky Performance Status; BM, brain metastases; EG, EGFR-TKI; Ch, chemotherapy; An, [antiangiogenic therapy](https://pubmed.ncbi.nlm.nih.gov/35842983/); Im, immunotherapy.

### Table S7 Baseline characteristics of T790M-positive and negative patients receiving immunotherapy.

| Baseline characteristic | T790M positive  (n=7) | T790M negative  (n=12) | P value |
| --- | --- | --- | --- |
| Age |  |  | 0.173 |
| <65 | 6 (85.7%) | 6 (50.0%) |  |
| ≥65 | 1 (14.3%) | 6 (50.0%) |  |
| Gender |  |  | 0.305 |
| Male | 3 (42.9%) | 2 (16.7%) |  |
| Female | 4 (57.1%) | 10 (83.3%) |  |
| Smoking history |  |  | 1.000 |
| No | 6 (85.7%) | 11 (91.7%) |  |
| Yes | 1 (14.3%) | 1 (8.3%) |  |
| Location |  |  | 0.350 |
| Right lung | 5 (71.4%) | 5 (41.7%) |  |
| Left lung | 2 (28.6%) | 7 (58.3%) |  |
| T stage |  |  | 0.245 |
| <2 | 0 (0.0%) | 4 (33.3%) |  |
| ≥2 | 7 (100.0%) | 8 (66.7%) |  |
| N stage |  |  | 1.000 |
| <3 | 4 (57.1%) | 8 (66.7%) |  |
| ≥3 | 3 (42.9%) | 4 (33.3%) |  |
| Other metastasis |  |  | 1.000 |
| No | 3 (42.9%) | 5 (41.7%) |  |
| Yes | 4 (57.1%) | 7 (58.3%) |  |
| KPS |  |  | 1.000 |
| <80 | 4 (57.1%) | 6 (50.0%) |  |
| ≥80 | 3 (42.9%) | 6 (50.0%) |  |
| BM number |  |  | 0.129 |
| <5 | 3 (42.9%) | 10 (83.3%) |  |
| ≥5 | 4 (57.1%) | 2 (16.7%) |  |
| BM symptom |  |  | 1.000 |
| No | 6 (85.7%) | 10 (83.3%) |  |
| Yes | 1 (14.3%) | 2 (16.7%) |  |
| EGFR mutation |  |  | 1.000 |
| Common | 6 (85.7%) | 11 (91.7%) |  |
| Uncommon | 1 (14.3%) | 1 (8.3%) |  |
| Combination with  EGFR-TKIs |  |  | 1.000 |
| No | 7 (100.0%) | 11 (91.7%) |  |
| Yes | 0 (0.0%) | 1 (8.3%) |  |
| Combination with  [antiangiogenic therapy](https://pubmed.ncbi.nlm.nih.gov/35842983/) |  |  | 0.656 |
| No | 2 (28.6%) | 5 (41.7%) |  |
| Yes | 5 (71.4%) | 7 (58.3%) |  |
| Combination with  chemotherapy |  |  | 1.000 |
| No | 2 (28.6%) | 4 (33.3%) |  |
| Yes | 5 (71.4%) | 8 (66.7%) |  |
| Combination with  radiotherapy |  |  | 1.000 |
| No | 6 (85.7%) | 11 (91.7%) |  |
| Yes | 1 (14.3%) | 1 (8.3%) |  |

Abbreviations: KPS, Karnofsky Performance Status; BM, brain metastases.

**Table S8** Univariable and multivariable cox proportional hazard regression analysis of risk factors potentially associated with OS1.

| Clinical Characteristics | Univariable analysis  p value | Multivariable analysis | | | |
| --- | --- | --- | --- | --- | --- |
|  |  | P value | Exp(B) | 95% CI | |
| Cohort  (A vs.B vs. C) | **0.000** | **0.000** | 2.260 | 1.889 | 2.704 |
| First-line therapy  (1,2 generation vs. 3 generation) | **0.001** | **0.035** | 0.741 | 0.561 | 0.979 |
| Age  (<65 vs.≥65) | 0.764 |  |  |  |  |
| Gender  (Male vs.Female) | **0.001** | 0.052 | 0.778 | 0.604 | 1.002 |
| Smoking history  (No vs.Yes) | 0.216 |  |  |  |  |
| Location  (Left lung vs.Right lung) | 0.709 |  |  |  |  |
| T stage  (<2 vs.≥2) | 0.812 |  |  |  |  |
| N stage  (<3 vs.≥3) | 0.920 |  |  |  |  |
| Other metastasis  (No vs.Yes) | **0.015** | 0.154 | 1.212 | 0.931 | 1.578 |
| KPS  (<80 vs.≥80) | **0.015** | **0.005** | 0.679 | 0.519 | 0.889 |
| BM number  (<5 vs.≥5) | **0.000** | **0.000** | 2.097 | 1.620 | 2.716 |
| BM symptom  (No vs.Yes) | 0.968 |  |  |  |  |
| EGFR mutation  (Common vs.Uncommon) | 0.075 |  |  |  |  |
| Subsequent treatment  (Continuation of EGFR-TKI or chemotherapy vs. Other treatment modalities) | **0.041** | 0.139 | 1.206 | 0.941 | 1.544 |

Abbreviations: Exp(B), odds ratio; CI, confidence interval; BM, brain metastases.
